# Commentationes Societatis Regiæ Scientiarum Gottingensis

**Published:** 1783

**Authors:** 


### THE LONDON MEDICAL JOURNAL,

For OCT. NOV. and DECEMBER, 1783.

### SECTION I.

### BOOKS.

I. *Commentationes Societatis Regiæ Scientiarum Gottingensis.* Classis Physicæ. Tomus III. ad A. 1780. 4to. Gottingæ, 1781. p. 148.

1. *DESCRIPTIONS of several rare and new plants cultivated in the Royal Botanic Garden. By* John Andrew Murray.—The plants described in this paper are, 1. Cercodia Erecta *Bankſii.* This plant, which constitutes a new genus, was first discovered by Sir Joseph Banks in New Zealand, and is now to be met with in all our botanic gardens. M. de Jussieu has given it the name of *Cunigunda,* and Spielmann in his catalogue of the botanic gar-

 den den at Strafburgh, printed in 1777, calls it *Cunigunda de Taiti*. 2. AQUILEGIA *nectariis rectis conniventibus petalo lanceolato æqualibus*. This is the *Aquilegia viridiflora* of Pallas, by whom the feeds were fent to our author. It is a native of Siberia. 3. GERANIUM *fcapis unifloris, foliis plerifque oblongis trilobis vel quinque-lobis incifo crenatis*. The author acknowledges himfelf indebted for this plant to Dr. Reichard of Franckfort, in honour of whom he names it *Geranium Reichardi*. 4. GERANIUM DAUCIFOLIUM *calycibus monophyllis foliis hirtis alternatim tripinnatis foliolis pinnatifidis*. Of this elegant plant, which has been neglected by modern botanifts, only an imperfect defcription is to be met with in Breyn and Rivinus, two fpecies of the fame genus having been confounded with each other. 5. OTHONNA TAGETES Linn. This plant is a native of the Cape of Good Hope, but has hitherto not been accurately defcribed. 6. PLANTAGO *foliis lineari fubulatis canaliculatis recurvatis punctatis denticulatis, fcapo tereti*. This is the *Plantago Incurvata* of Juffieu. 7. HYPERICUM *floribus trigynis, primordialibus feffilibus, caule ancipiti fruticofo, foliis lanceolato linearibus* LINN. *Syft. Veg.* p. 583. The defcriptions of this this and the other plants are illuftrated with good engravings.

11. *Chirurgical obfervations.* *By* Aug. Gottl. Richter.—Six cafes are related in this paper. The firft is an inftance of amaurofis after the fmall pox, the cure of which feems to have been effected chiefly by the ufe of fternutatories. The fecond is a cafe of a fractured thigh, in which an extended pofition of the limb was found to be neceffary. The third is a cafe of obftructed vagina in a girl twenty years old. For the fpace of three years fhe had complained of violent periodical pains about the os facrum, accompanied with rigors, anxiety, and fometimes with *deliquium animi.* Thefe painful fymptoms had returned every month, but without any appearance of the catamenia. On examination the vagina was found clofed at its upper part, and by introducing a finger of one hand into the vagina, while a preffure was made on the abdomen with the other, a fluctuation was perceptible above the ftricture. On inquiry it was found that the patient had had a confluent fmall pox when fhe was thirteen years old, and that the difeafe had been followed with a painful difcharge of pus from the vagina. The obftruction

ſeemed therefore to have originated from a variolous ulcer.

The ſtricture having been removed by a ſurgical operation, a conſiderable quantity of blood was evacuated, which was not in the leaſt degree fœtid. The vagina was kept open by means of ſuppoſitories, the wound healed without difficulty, and the patient has ſince had a regular diſcharge of the menſes, and enjoyed good health.

In the fourth obſervation, we have an account of the extirpation of a cancer of the lower lip, in which we meet with ſome uſeful cautions concerning the means of bringing together the lips of the wound in ſuch caſes.

The fifth obſervation contains three inſtances of *Ptoſis* or fall of the eyelids. The ſubject of the firſt of theſe caſes was a peaſant, afflicted with a periodical complaint of this ſort, which returned at irregular intervals. Smoking tobacco never failed to bring it on, and the patient obſerved, that by putting on a pair of ſpectacles he inſtantly got rid of the paroxyſm, and prevented its return, ſo that at laſt he ſeldom went without them. After cleanſing the primæ viæ, our author adminiſtered aſſafœtida and valerian, and at the ſame time directed a bliſter to be applied to the patient's forehead near his eye-brows.

After this the complaint never returned. In the second case the disease was occasioned by an injury done to the musculus levator palpebræ superioris, some of the fibres of which were lacerated, so that the patient was unable to raise that portion of the eye-lid. The disease was cured by dividing the lacerated fibres with a knife. In the third instance, the complaint was purely spasmodic. The patient was a young woman twenty years of age, who was subject to occasional spasms not only of her eye-lids but of the other muscles of her face. In this case the affection gave way to musk and small doses of emetic tartar.

In the sixth section, the author treats of depressions of the cranium in young subjects.

III. *Experiments relative to the dying of Cotton with the flowers of Carthamus* (Bastard Saffron) *by* John Beckmann.

IV. *Remarks on Argillaceous Earths, and more particularly on a species of clay found in the neighbourhood of Urach. By* John Fred. Gmelin.

V. *Remarks on the fundamental meteorological year**. *By* John Christoph. Gatterer.—This elaborate essay claims the attention of all who de-

* De anno meteorologico fundamentali commentatio.

vote vote their time to meteorological obfervations. If it fhould be afked, fays the author, why I adopt the term *fundamental*, it is becaufe the obfervations I have made in the courfe of one year will ferve as a bafis for fimilar refearches in future, not only at Gottingen but in every other part of the world. He defcribes his plan, and exhibits a fketch of fome of the tables he has formed. He begins with his *meteorological folar tables*, in which he notices the variations of the folar equator and axis, the diminution or increafe of the obliquity of the ecliptic, the inequality of the fun's light, &c. Next follow his *meteorological lunar tables*, which are eight in number, and thefe are fucceeded by nine others called *comparative meteorological tables*, in which the united effects of the fun and moon are noticed. The author then defcribes his *terreftrial or local meteorological tables*, in which he attends to local winds; the diminution of heat in proportion to the elevation of places above the furface of the ocean; the retardation or acceleration of meteors, according to the diftance of places from the fea; the drynefs or moifture of a country; variations of atmofpherical electricity; thunder and lightning; variations of the magnetic needle; and laftly, the meteorological fitua-

ſituation of Gottingen. He then gives an account of his *auxiliary meteorological tables*, which contain comparative tables of different barometers and thermometers, &c. Theſe are followed by obſervations on meteorological predictions (*vaticinia meteorologica*), and the eſſay cloſes with directions for compoſing a meteorological calendar, illuſtrated with tables.

VI. *An account of the Aptenodytes, a genus of birds found only in the ſouthern ocean. By* John Reinhold Forſter, L.L.D.

